# A complex behavioural change intervention to reduce the risk of diabetes and prediabetes in the pre-conception period in Malaysia: study protocol for a randomised controlled trial

**DOI:** 10.1186/s13063-016-1345-x

**Published:** 2016-04-27

**Authors:** Jutta K. H. Skau, Awatef Binti Amer Nordin, Julius C. H. Cheah, Roslinah Ali, Ramli Zainal, Tahir Aris, Zainudin Mohd Ali, Priya Matzen, Regien Biesma, Jens Aagaard-Hansen, Mark A. Hanson, Shane A. Norris

**Affiliations:** MRC Developmental Pathways for Health Research Unit, Department of Paediatrics, School of Clinical Medicine, Faculty of Health Sciences, University of the Witwatersrand, Johannesburg, South Africa; Institute of Health System Research, Ministry of Health, Selangor, Malaysia; School of Medicine and Health Sciences, Monash University Malaysia, Jalan Lagoon Selatan, 46150 Bandar Sunway, Selangor, Malaysia; Pharmaceutical Policy and Strategic Planning Division, Pharmaceutical Services Division, Ministry of Health Malaysia, Selangor, Malaysia; Institute of Public Health, Ministry of Health, Kuala Lumpur, Malaysia; State Health Department Negeri Sembilan, Ministry of Health, Seremban, Malaysia; Institute of Developmental Sciences, Faculty of Medicine, University of Southampton, Tremona Road, Southampton, SO16 6YD UK; Department of Epidemiology and Public Health Medicine, Royal College of Surgeons in Ireland, Dublin, Ireland; Health Promotion Research, Steno Diabetes Center, Gentofte, Denmark

**Keywords:** Pre-conception, Pre-pregnancy, Complex behavioural change intervention, E-health, Malaysia, Lifestyle intervention, Motivational counselling

## Abstract

**Background:**

Over the past two decades, the population of Malaysia has grown rapidly and the prevalence of diabetes mellitus in Malaysia has dramatically increased, along with the frequency of obesity, hyperlipidaemia and hypertension. Early-life influences play an important role in the development of non-communicable diseases. Indeed, maternal lifestyle and conditions such as gestational diabetes mellitus or obesity can affect the risk of diabetes in the next generation. Lifestyle changes can help to prevent the development of type 2 diabetes mellitus. This is a protocol for an unblinded, community-based, randomised controlled trial in two arms to evaluate the efficacy of a complex behavioural change intervention, combining motivational interviewing provided by a community health promoter and access to a habit formation mobile application, among young Malaysian women and their spouses prior to pregnancy.

**Method/design:**

Eligible subjects will be Malaysian women in the age group 20 to 39 years, who are nulliparous, not diagnosed with diabetes and own a smartphone. With an alpha-value of 0.05, a statistical power of 90 %, 264 subjects will need to complete the study. Subjects with their spouses will be randomised to either the intervention or the control arm for an 8-month period. The primary endpoint is change in waist circumference from baseline to end of intervention period and secondary endpoints are changes in anthropometric parameters, biochemical parameters, change in health literacy level, dietary habits, physical activity and stress level. Primary endpoint and the continuous secondary endpoints will be analysed in a linear regression model, whereas secondary endpoints on an ordinal scale will be analysed by using the chi-squared test. A multivariate linear model for the primary endpoint will be undertaken to account for potential confounders. This study has been approved by the Medical Research and Ethics Committee of the Ministry of Health Malaysia (protocol number: NMRR-14-904-21963) on 21 September 2015.

**Discussion:**

This study protocol describes the first community-based randomised controlled trial, to examine the efficacy of a complex intervention in improving the pre-pregnancy health of young Malaysian women and their spouses. Results from this trial will contribute to improve policy and practices regarding complex behavioural change interventions to prevent diabetes in the pre-conception period in Malaysia and other low- and middle-income country settings.

**Trial registration:**

This trial is registered with ClinicalTrials.gov (www.clinicaltrials.gov) on 30 November 2015, Identifier: NCT02617693.

**Electronic supplementary material:**

The online version of this article (doi:10.1186/s13063-016-1345-x) contains supplementary material, which is available to authorized users.

## Background

Diabetes mellitus (DM) is one of the most common non-communicable diseases (NCDs) across the world. Today 387 million people are living with diabetes whereas 46.3 % remain undiagnosed. The prevalence of DM is expected to increase to 53 % by 2035 [1]. DM is an important public health concern in Malaysia. Over the past two decades, the population of Malaysia has grown rapidly and the prevalence of DM in Malaysia has increased dramatically, along with the prevalence of obesity, hyperlipidaemia and hypertension. The National Health and Morbidity Survey (NHMS) 2015 estimated the prevalence of DM in adults 18 years old and over was 11.6 % in 2006 and 17.5 % in 2015 [2]. The prevalence of DM has almost tripled over the past two decades in the younger generations (20–39 years old) [2, 3].

There is a body of evidence showing that lifestyle changes, such as achieving a healthy body weight, can help to prevent the development of type 2 diabetes mellitus (T2DM) [4–6]. Complex behavioural change interventions, or lifestyle intervention trials, have also moved in the past decade from targeting high-risk to low-risk populations and interventions have also been targeted earlier in the life course [7–9]. The use of new communication technologies, such as computer-based interventions, and empowerment techniques such as goal setting, have been found to be helpful and eventually more effective than other interventions on weight loss and self-management of T2DM [10–13]. Lifestyle interventions seek to delay or halt the development of T2DM and achieve, e.g. a healthy body weight. Goal setting requires a coordinated team effort that involves the person at risk in the decision-making process. Evidence demonstrates that structured, intensive lifestyle programmes involving participant education, individualised counselling, reduced dietary energy and fat intake, regular physical activity, and frequent participant contact are necessary to produce long-term weight loss of 5–7 % [14].

The Developmental Origins of Health and Disease (DOHaD) concept describes how during early life (at conception, and/or during foetal life, infancy and early childhood), the environment induces changes in development that have a long-term impact on later health and disease risk [15]. Early-life influences play an important role in the development of NCDs, and maternal lifestyle and conditions such as gestational diabetes mellitus (GDM) or obesity can affect the risk of diabetes in the next generation [16, 17]. Besides complications of pregnancy such as GDM, hypertension, and preeclampsia that directly affect the pregnancy outcome, the mother’s health during pregnancy affects her child’s risk of becoming overweight or obese. If the mother makes lifestyle choices that lead to unbalanced nutrition, obesity, stress, smoking and others, her child is more vulnerable to diabetes and other NCDs [18]. Children born to mothers with GDM have a four to eight times higher risk of obesity, glucose intolerance and T2DM in adolescence or young adulthood [19].

The Jom Mama project has been designed to develop an intervention which responds to this rising public health challenge, focusing on young Malaysian women and their spouses. The overall aim of the Jom Mama project is to demonstrate whether a complex behavioural change intervention will change markers of health in the young Malaysian woman in the pre-conception period, before evaluating whether the intervention affects the pregnancy course and the neonate susceptibility to obesity and diabetes. The Jom Mama project is a public-private partnership between the Ministry of Health (MoH), Malaysia; the University of Southampton, UK; the University of Witwatersrand, South Africa; the Steno Diabetes Centre, Denmark; and Novo Nordisk, Denmark. The project was initiated in 2012 and is following Intervention Mapping guidelines, which is a systematic framework to develop health promotion programmes [20]. The Jom Mama project is conducted in three phases; phase 1: to develop knowledge on the lifestyle of young couples in Malaysia and identify the barriers and facilitators of health [18, 21]; phase 2: to develop intervention matrices and develop an intervention package based on the outcome from phase 1; and phase 3: to implement and evaluate the developed intervention package in a randomised control trial (RCT). In this paper we will present the study protocol for an unblinded, community-based, RCT with two arms to evaluate the complex behavioural change intervention developed in phase 2 of the Jom Mama project.

## Methods/design

### Aim

The primary objective of the trial is to evaluate the efficacy of a complex behavioural change intervention combining behaviour change counselling provided by community health promoters (CHPs) and utilisation of an E-health platform to enhance women’s health prior to pregnancy. This will be measured through the change in waist circumference after the 8-month intervention period between female subjects exposed to the intervention and female subjects exposed to standard of care in a control group. The secondary objectives are to evaluate the efficacy of the intervention on health literacy, dietary habits, physical activity, sedentary behaviour and stress level.

### Study design

**Fig. 1:**
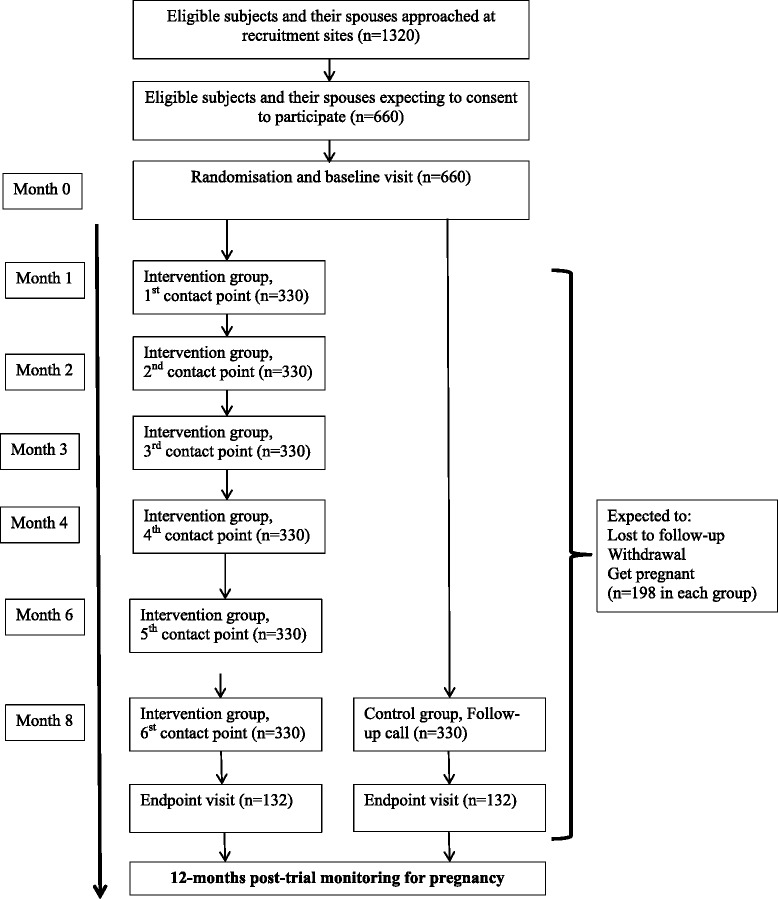
The flow diagram of the Jom Mama trial

The trial is designed as an unblinded, RCT to assess the efficacy of a complex behavioural change intervention aiming to improve the overall health of young women prior to their first pregnancy. The intervention follow-up period is 8 months from randomisation/baseline. After end trial the subjects will be monitored for a period of 12 months for pregnancy. Figure 1 gives the flow diagram of the trial. The trial was designed following the Standard Protocol Items: Recommendations for Interventional Trials (SPIRIT) 2013 statement (see Additional file 1).

### Site and participation selection

The trial will be carried out in the district of Seremban in the state of Negeri Sembilan, Malaysia. This location was selected due to Negeri Sembilan having a higher prevalence of diabetes (22.0 %) compared to the national prevalence (15.2 %) [22]. Ethnic diversity is one of the key features of Malaysia and the district of Seremban has a representation of the three major ethnic groups: Malay, Chinese and Indian, 67.4, 24.6 and 7.3 %, respectively [23].

The target subjects will be newly registered married or engaged women. The woman will be recruited with her spouse, who also will be exposed to the intervention.

### Inclusion and exclusion criteria

Eligible subjects must meet all the following inclusion criteria:

1. Female and between the ages of 20–39 years
2. Nulliparous
3. Not pregnant at the time of signing the informed consent
4. Own a smartphone, with either an Android operating system version 4.1 and above or iOS operating system 7.0 and above, and have Internet access

Subjects meeting any of the following criteria will be excluded from participation in the trial:

1. Female subject undergoing treatment for type 1 or 2 diabetes mellitus
2. Subject not residing in the district of Seremban

### Screening

Newly married or engaged women and their spouses will be recruited from 2 November 2015, primarily from five designated primary health clinics in the district of Seremban, whereas Muslim Malay couples are obligated to conduct a pre-marriage screening, which involves a HIV/AIDS test. Other sites in Seremban such as the state marriage registration office, temples and churches or other sites (work places, malls, gyms, etc.) will also be approached for recruitment purposes. A research officer will meet the young couples face-to-face and provide an explanation of the study rationale and a description of the Jom Mama project. The women and spouse will be given an opportunity to ask questions and those who are interested in the study will have their contact details recorded for further contact and arrangements for a baseline visit. If possible, the couple will be given a clinic appointment for baseline measurements at one of the five designated primary health clinics in Seremban. If the couple need time to consider their involvement, the research officer will follow-up with three phone calls over the next 3 weeks. If, at these phone calls the couple accepts participation in the trial, a baseline appointment will be made. If the couple does not answer the three phone calls or rejects the offer to participate, they will be dropped from the participation registration list.

### Intervention

**Fig. 2:**
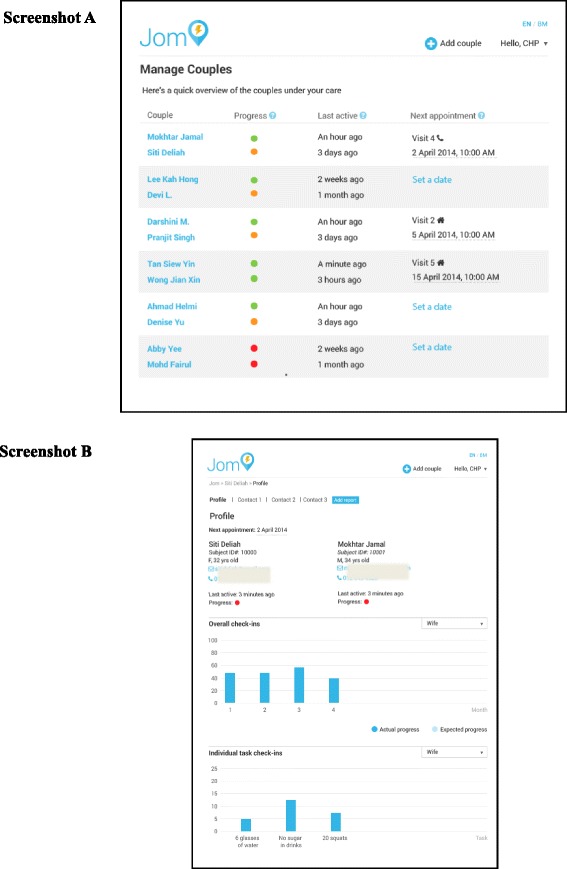
Screen shot from the web application, showing the couples’ dashboard. Screen shot **a** Shows the overview of all couples under one community health promoter (CHP). The traffic lights give an indication on how active the couple are in performing their selected challenges. The *second column* indicates when the couple was last active on the mobile application. The *last column* informs when the CHP has the next appointment with the couple. Screen shot **b** Shows the progress over time for one couple. The graphic can shift between wife and husband and, in this example, it is only showing the wife. The *upper columns* indicate how many times the challenges have been performed per month. The *lower columns* indicate how many times each challenge has been performed

The intervention has two components. The first component is an interaction with a CHP who will be in contact with the women and their spouses during the 8-month intervention period. These contacts will be a combination of three face-to-face meetings, three phone calls and communication through Whatsapp group chat. The CHP will use motivational counselling techniques, i.e. motivational interviewing, to support and motivate the young women and their spouses to live a healthier lifestyle. The second component is an E-health platform which consists of two elements: (1) a mobile application in the form of a habit formation application, where subjects and spouses can select different challenges for obtaining a healthy diet, more physical activity and less sedentary behaviour during the intervention period; and (2) a web-based back-end interface which can be accessed by the CHP, which will be her tool to assess the women and their spouses’ lifestyle situation. The CHP uses a dashboard to follow the progress of subject and spouse in performing the challenges, and uses the information to interact with the subject and her spouse during the contact points in the intervention. Figure 2 presents screen shots of the web-based application the CHPs are using. The CHP will function as a personal coach to engage, support and guide the young woman and her spouse toward achieving and maintaining a healthier lifestyle. During the interaction over the intervention period of 8 months, the CHPs will assess the subject and her spouse’s health risks and behaviour, share information on healthy living and support the subjects and spouses to reach their personal health goals. The objectives of each contact point are presented in Table 1.

**Table 1:** Overview of the objective of each face-to-face and phone call contact point

Contact point	Objective
Contact point 1 (face-to-face)	Assessment of woman and spouse’s health risk and behaviour: conversation starter
	Introduce to use of mobile application
	Initiate review of assessments and behaviour change process
	Goal setting
Contact point 2 (face-to-face)	Follow-up on use of mobile application
	Provide health information as required
	Review and address extrinsic supportive and inhibiting factors
	Introduce proactive coping
Contact point 3 (phone)	Follow-up on use of mobile application
	Follow-up on progress based on initial goal setting
	Provide support and advice
	Provide health information as required
	Revise goal setting and set new goals as relevant
Contact point 4 (phone)	Follow-up on use of mobile application
	Follow-up on progress based on previously set goals
	Provide support and advice
	Provide health information as required
	Revise goal setting and set new goals as relevant
Contact point 5 (face-to-face)	Follow-up on use of mobile application
	Follow-up on progress based on previously set goals
	Provide support and advice
	Provide health information as required
	Revise goal setting and set new goals as relevant
Contact point 6 (phone)	Follow-up on progress based on previously set goals
	Provide support and advice
	Provide health information as required
	Goal setting and planning for the future
	End of intervention process

The CHP role will be carried out by community nurses who are currently working in primary health care in Malaysia. Prior to the start of the intervention, the community nurses undertook training in becoming a CHP. This was a 5-day training programme with sessions in motivational interviewing techniques, the use of the E-health platform and basic knowledge of healthy diet and physical activity. The training was carried out by local trainers who are social-psychologists with expertise in motivational interviewing techniques, professional dieticians and physical activity experts. During the trial, refreshment training with the social-psychologist trainers will take place to support and help the CHPs in addressing challenging situations they encountered with the young women and their spouses.

**Fig. 3:**
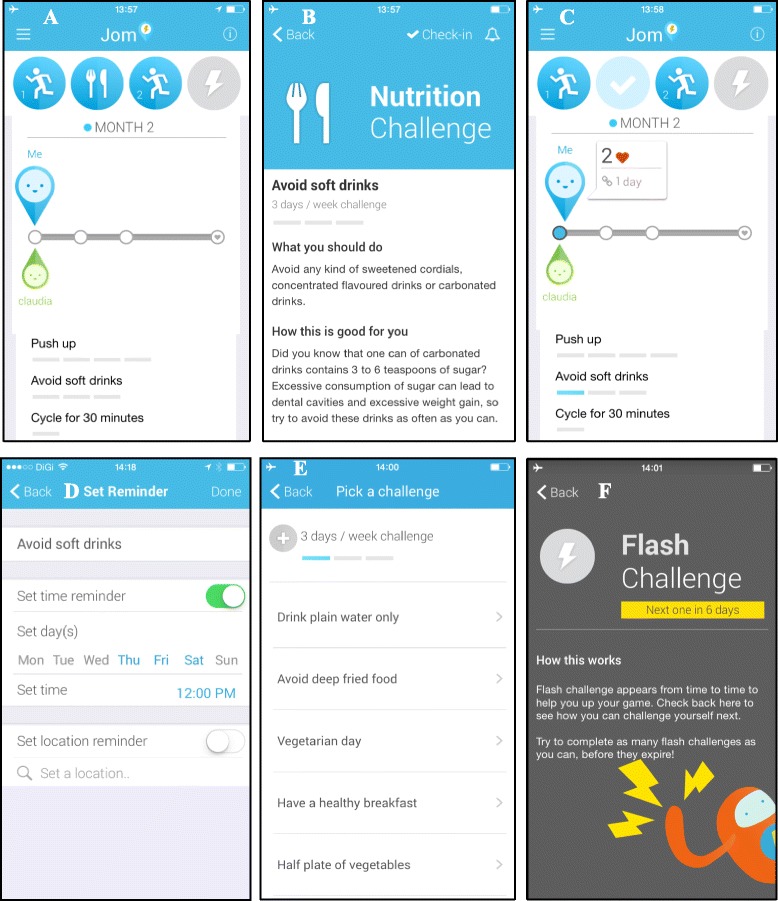
Screen shots of mobile application. **a** The main screen, showing the couple’s process for the particular month. The icons on *top* illustrate the categories of the different challenges selected (two physical activity challenges and one healthy eating challenge). In the *middle*, is the progress bar, showing the progress of the user and her spouse for this particular month. *Lowest*, are the selected challenges listed and how many times they should be performed in one week. **b** By tapping on the challenges, a description of the different challenge is showing (example of the healthy eating challenge). **c** When the challenge has been performed, the subject will ‘check-in’, and by doing that be awarded 2 points for performing the challenges. **d** A timer can be set for each challenge, so a reminder will be send to the subject on the given time to perform the challenge. **e** The subject is selecting the challenge out of a list set challenges. **f** The Flash challenge is a bonus challenge, which the subject can select when it becomes available. This occurs randomly during the intervention period. The Flash challenge will give extra points

The mobile application is a habit-formation application, which incorporates personal goal setting, progress tracking and general information on healthy lifestyles. The mobile application has two key components comprising diet and physical activity. Under each domain several challenges have been developed which can be selected by the subject on a monthly basis in dialogue with the CHP. The application will suggest several challenges for healthy eating, such as making healthy food choices and healthy food preparation, and interactive options for the subject to stay active and prevent sedentary behaviour. In addition, the mobile application will also provide information on what constitutes a healthy lifestyle and further information on how to stay healthy before pregnancy. The mobile application is available on iOS and Android operating systems. Figure 3 presents several screen shots from the mobile application.

### Control arm

Subjects in the control arm will receive standard of care, which is no contact with a CHP and no access to the E-health platform. They will receive one phone call from a research officer towards the end of the intervention period to remind them of their endpoint visit.

### Outcomes

#### Primary endpoint

Change in waist circumference was selected as the primary endpoint because of its validity as a superior measurement for assessing abdominal fat content and associated obesity health risk, as compared to a measure such as body mass index (BMI) [24–26]. In addition, there are existing national Malaysian data on population waist circumference, which allow for the study results to be comparable on a national level. In the present trial, the primary endpoint is predicted to provide a 2-cm reduction of waist circumference in the intervention group compared to the control group, which will reflect a reduction of central fat distribution. The reduction of 2 cm in waist circumference as a primary endpoint is based on the mean from a number of studies indicating that a decrement of waist circumference is possible with a lifestyle changing intervention over a period of 8 months [27–31]. The variation between the two time-points in these studies was small (standard deviation (SD) of 3–5) after the intervention period, and we therefore assume an expected within-person estimate of SD = 5.

#### Secondary endpoints

The secondary endpoint will be as follows:

1. Change in BMI from baseline to after 8 months
2. Change in waist-to-height ratio from baseline to after 8 months
3. Change in waist-to-hip ratio from baseline to after 8 months
4. Change in weight from baseline to after 8 months
5. Change in glycated haemoglobin A_1c_ (HbA_1c_) from baseline to after 8 months
6. Change in the fasting lipid profile (total cholesterol, low-density lipoprotein cholesterol, high-density lipoprotein cholesterol and triglycerides) from baseline to after 8 months
7. Change in blood pressure (systolic and diastolic blood pressure) from baseline to after 8 months
8. Change in the level of health literacy, as measured by the European Health Literacy Survey Questionnaire (HLC-EU-Q47) from baseline to after 8 months
9. Change in dietary intake, as measured by a Food Frequency Questionnaire (FFQ), from baseline to after 8 months
10. Change in physical activity and sedentary behaviour, as measured by the International Physical Activity Questionnaire (IPAQ) used in national health surveys in Malaysia, from baseline to after 8 months
11. Change in stress levels, as measured by the Depression Anxiety and Stress Scale 21-items (DASS-21) used by general practitioners in the Malaysian primary health care system from baseline to after 8 months

#### Other endpoints

The incidence of GDM, confirmed by an oral glucose tolerance test (OGTT), will be assessed in the subjects who will complete the intervention period and who become pregnant in the post-trial monitoring period. The OGTT will be taken at weeks 26–28 of the pregnancy. Data will be captured retrospectively and obtained from medical charts.

#### Supportive data

Anthropometric measurements, blood pressure and data on physical activity, dietary, stress and health literacy will be collected from the subject’s spouse. Socio-demographic data and family medical history will be collected from both the subject and the spouse. These variables are collected to adjust for confounding effects.

#### Sample size calculation

The sample size calculation is based on a 2-cm reduction in waist circumference, in the primary endpoint of waist circumference between the intervention and control arms at 8 months. Assuming a 5 % level of significance (alpha = 0.05), a statistical power of 90 % (beta = 0.10) and a SD of 5 cm using a two-sided *t* test, 132 women per arm are required. Taking into consideration a 20 % drop-out rate, and that 20 % will be pregnant, a total of 660 women would be randomised/enrolled to ensure 264 subjects to cover both the intervention and control arms.

#### Randomisation

At the baseline measurement visit, subjects will be randomised into the intervention and control arms at a 1:1 allocation ratio. Random allocation sequences will be computer- generated with block sizes of six subjects. Each clinic will be provided with a sufficient number of identification (ID) numbers with randomisation codes. When a subject is enrolled in the trial, the study nurse should assign the lowest available ID number to the subject from the list of ID numbers.

The Institute for Health System Research (IHSR) under the MoH has prepared the randomisation list and these are distributed to the five designated primary health clinics in Seremban. A copy is maintained by the IHSR.

### Data collection, management and analysis

#### Study conduct

Study nurses will be trained to conduct the data collection at the five designated primary health clinics in Seremban district. At the baseline assessment, the principles of the trial will be explained again for the subject and spouse and they will be asked to provide consent by signing the consent form. One original copy of each form will be kept by the study nurse who will file the document on site. One copy of each form will be given to the subjects. The women and their spouses will have linked ID numbers by assigning each subject and spouse the same ID number with subject ID ending with the number ‘2’ and spouse ID ending with the number ‘1’ respectively.

#### Data collection

At baseline the subjects will undergo measurements of waist circumference, hip circumference, weight, height and blood pressure by following standardised procedures based on World Health Organisation (WHO) STEPS surveillance [32]. Only the subject in the couple will provide a 10-mL blood sample to measure glycated haemoglobin A_1c_ (HbA_1c_) and a fasting lipid profile (triglycerides (TG), low-density lipoprotein cholesterol (LDL-C), high-density lipoprotein cholesterol (HDL-C) and total cholesterol (TC)). Due to the need for a fasting lipid profile analysis, the subject will be asked to come to the clinic after fasting for a minimum of 9 h. In line with this, the data collection time-points are planned to be in the morning. The European Health Literacy Tool Q-47 (HL-EU-Q47) [33] (HL-EU Consortium 2012), the Malaysian Food Frequency Questionnaire (FFQ), modified to be used in the current trial [2]; the International Physical Activity Questionnaire (IPAQ) [2]; and the Depression Anxiety Stress Scale 21-items (DASS-21) [34, 35] will be used to collect data on level of health literacy, dietary pattern, physical activity level, and stress level, respectively. Data such as socio-demographic parameters and family history of diabetes will be collected by structured interviews with the study nurse. The spouse will undergo the same data measurements, with the exception of blood sampling. At the end of the data collection, and before subject and spouse leave, data will be checked by the study nurse and site coordinator or research officer, to ensure that all data forms and questionnaires have been filled correctly. When data collection is completed the data collection forms and questionnaires will be signed-off by the study nurse and site coordinator or research officer. At the end of the baseline visit, if the subject is randomised to the intervention, the first contact with a CHP will be made. The subject and spouse will be introduced to the mobile application and it will be installed on the subject’s and spouse’s smartphone device. If the subject and spouse have sufficient time the CHP will proceed with the first contact point. If the subject and spouse do not have the time, an appointment will be set in the next 5 days either at the clinic or at the couple’s house. The same measurements, except height, socio-demographic parameters and medical history, described above will be conducted at the endpoint visit (Table 2).

**Table 2:**
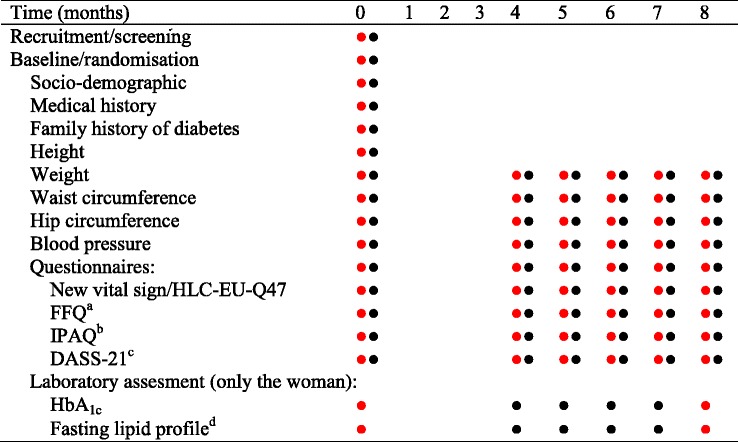
Data collection at each time-point in the Jom Mama trial

Data collection on woman;● Data collection on spouse; ^a^ *FFQ* Food Frequency Questionnaire, ^b^ *IPAQ* International Physical Activity Questionnaire, ^c^ *DASS*-*21* Depression Anxiety and Stress Scale 21-items, ^d^ Fasting lipid profile: will be measured as a combination of: total cholesterol (TC) level, low-density lipoprotein cholesterol (LDL-C) level, high-density lipoprotein cholesterol (HDL-C) level and triglyceride (TG) level;  ^5^Exit point visit will occur if the woman becomes pregnant between 4.1 months and 8 months after randomisation

If a subject becomes pregnant within the 8-month intervention period, she will be withdrawn from the trial. Exit measurements, which are similar to those at the endpoint visit, will be performed only for subjects who become pregnant between 4.1 and 8 months from baseline. For those who become pregnant between baseline and month 4 in the intervention period, no measurements will be taken.

#### Data management

All data will be managed and stored with protection of data privacy. Documentation as well as corrections, if necessary, will be done in accordance to Good Clinical Practice guidelines. All protocol-required information collected during the trial must be entered on a data collection form containing the four questionnaires. The original completed data collection form will be send to IHSR, MoH Malaysia, where double data entry will be performed by two independent data clerks. The completeness, validity and plausibility of data are examined by validating programmes, which thereby generate queries. The generated queries will be checked with original data collection forms and all changes from the original data are documented on audit files. Data will be maintained and analysed in the statistical Stata programme, version 12.0 or higher (StataCorp LP). Monitoring will be performed regularly by an independent clinical research organisation from Malaysia to check the completeness of the contents of records and the data collection forms, the adherence to the protocol and the progress of enrolment.

#### Statistical analysis

The null hypothesis is that there is no difference in the change in waist circumference between the intervention and the control group after the 8-month intervention. The alternative hypothesis is that the decrement in waist circumference will be bigger in the intervention group compared to the control group after the 8-month intervention. A linear regression model will be used to assess the difference in the change of waist circumference from baseline to end of intervention, with the difference in waist circumference as the independent variable, treatment (intervention versus control) and baseline waist circumference as covariates. Additional tests will be performed for the impact of the intervention in both high- (waist circumference above 80 cm) and low-risk (waist circumference below 80 cm) groups. This will be done by subgroup analysis or by assessing the proportional changes in the primary endpoint. Each subgroup will also be analysed in a linear regression model. The continuous secondary endpoints will be analysed in a linear regression model, whereas the difference in the change of each secondary endpoint from baseline to end of intervention, will be the dependent variable, treatment (intervention versus control) and baseline value as covariate. Regarding health literacy (HL-EU-Q47), physical activity (IPAQ) and stress (DASS-21) all the variables are ordinal and, therefore, independence between intervention and control groups will be tested with the chi-squared test. Change in nutrient intakes will be compared between the groups using a generalised linear model, with additional adjustments for total energy intake. Nutrient and food patterns before and after the intervention will be explored in both groups separately by using dimension reduction techniques such as principal component analysis. Multivariate analysis will be performed to assess the association between exposure to the intervention, primary endpoints and secondary endpoints (BMI, waist-to-height and waist-to-hip ratio, HbA_1c_, lipid profile, blood pressure, health literacy, physical activity, dietary intake and stress level) using linear regressions. In this model the secondary endpoint will be included in the model as covariates and the estimated coefficients will be presented unadjusted and adjusted for appropriate and/or effect modifier and/or confounders. Furthermore, interactions between the secondary endpoints will also be explored. Several supportive analyses will be performed. The first supportive analysis will explore the relation between subjects’ and spouses’ change in waist circumference and other anthropometric measurements, diet, physical activity, health literacy and stress. A second supportive analysis will explore whether a short intervention period (subjects who become pregnant between 4.1 and 8 months in the intervention period), will also have an impact on the primary endpoint and secondary endpoints. A time variable explaining how long they have been included in the intervention will be included in the models as a covariate. At the end of the 12-month post-intervention monitoring period, a full analysis will be conducted on subjects who become pregnant within 12 months after completing the intervention. This will be done by assessing the incidences of GDM among women who became pregnant between treatment groups, using a chi-squared test. Additionally, a logistic regression model to explore associations between the number of subjects with GDM and several secondary endpoints will be conducted.

#### Ethical, regulatory and data management considerations

The study protocol version 4.0 dated 12 August 2015 has received ethical and governance approvals by the Medical Research and Ethics Committee of the Ministry of Health Malaysia (protocol number: NMRR-14-904-21963) on 21 September 2015. The study is performed in compliance with the Declaration of Helsinki. Written informed consent will be taken from each participant prior to study enrolment by trained study nurse. The trial was registered at ClinicalTrials.gov, Identifier: NCT02617693 on 30 November 2015.

#### Safety monitoring

In the event of an abnormal laboratory test results for Hb1Ac or a newly suspected or diagnosed health concern during a subject’s measurements (i.e. high blood pressure or a high score in the DASS-21 tool) at baseline/exit point/endpoint, the subject will be referred to their primary health care physician for further examination.

## Discussion

This study protocol describes the first community-based RCT to examine the efficacy of a complex behavioural change lifestyle intervention in improving the pre-pregnancy health of young Malaysian couples. Other studies assessing the prevention of GDM and T2DM have mainly focused on high-risk groups or the intervention entry point has been in the first trimester of the women’s pregnancy [36–40], whereas few RCT studies exist on low-risk populations or on an intervention entry point in the pre-conception period [41]. To develop population strategies, instead of targeting high-risk groups it is imperative to widen the focus to the general population, especially in a country such as Malaysia where the prevalence of diabetes and obesity has increased alarmingly in the last 10 years [2]. One European study has shown that limited guidelines and policies exist in this area, as guidelines and policies are designed to mainly target high-risk groups [42]. Therefore, this study may contribute evidence to this important area which is needed to support policy-makers in making rational decisions to improve population health.

A pilot test was conducted prior to trial implementation and has identified a number of challenges. A major challenge that has been found is that the intervention addresses a challenging period for young Malaysian couples, whereby busy work schedules and lack of time affect participation rates and generate low interest in such an intervention. While it would be ideal to collect spousal data at baseline and endpoint, it will be made optional for the spouse to attend both baseline and endpoint visits if they are unable to attend in order to continue with recruitment. Based on these observations, it is expected that the recruitment period will be longer than initially anticipated.

The combination of motivational interviewing through a CHP and E-health platform is a novel approach. Training the community nurses to become CHPs involves an interactive approach beyond their conventional training programmes; this provided new learning experiences for these nurses to build higher capacity in patient engagement. The E-health platform is also a new experience for the primary health care services in the Seremban district and considerable time was needed to train community nurses with the necessary IT skills. Another key challenge faced was the limited usage of the E-health mobile application due to poor Internet connectivity in the district of Seremban. In mitigating this challenge, Wi-Fi hotspots in the designated primary health clinics have been set up to enable participating women and their spouses to download the mobile application in a secured Internet environment. Additionally, several changes have been made to the E-health platform to make it more robust.

The proposed trial will not directly assess the long-term effects of the intervention on the risk of the offspring developing T2DM. Given the complexity of developing, implementing and evaluating complex behavioural change lifestyle interventions, this trial will focus on improving the pre-conception health in young women prior to their first pregnancy.

## Trial status

The trial started recruitment in November 2015. The trial is expected to be completed by July 2017.

## Additional file

Additional file 1: The SPIRIT recommendations for clinical trials protocols. (DOC 117 kb)

## Abbreviations

BMI: body mass index
CHP: community health promoter
DASS-21: Depression Anxiety and Stress Scale 21-items
DBP: diastolic blood pressure
DM: diabetes mellitus
DOHaD: The Development Origins of Health and Disease
FFQ: Food Frequency Questionnaire
GDM: gestational diabetes mellitus
HbA_1c_: glycated haemoglobin A_1c_
HDL-C: high-density lipoprotein cholesterol
HLC-EU-Q47: The European Health literacy Survey Questionnaire
ID: identification
IHSR: Institute for Health System Research
IPAQ: International Physical Activity Questionnaire
LDL-C: low-density lipoprotein cholesterol
MoH: Ministry of Health
NCDs: non-communicable diseases
NHMS: National Health and Morbidity Survey
OGTT: oral glucose tolerance test
RCT: randomised controlled trial
SBP: systolic blood pressure
SD: standard deviation
T2DM: type 2 diabetes mellitus
TC: total cholesterol
TG: triglycerides
WHO: World Health Organisation

## References

[1] International Diabetes Federation (IDF) (2014). IDF Diabetes Atlas.

[2] Institute for Public Health (IPH) (2015). National Health and Morbidity Survey 2015 (NHMS 2015). Vol. II: non-communicable diseases, risk factors and other health problems.

[3] Institute for Public Health (IPH) (1996). National Health and Morbidity Survey 1996 (NHMS 1996). Vol. II: non-communicable diseases.

[4] Ivy JL (1997). Role of exercise training in the prevention and treatment of insulin resistance and non-insulin-dependent diabetes mellitus. Sports Med..

[5] Tuomilehto J, Lindstrom J, Eriksson JG, Valle TT, Hämäläinen H, Ilanne-Parikka P, Finnish Diabetes Prevention Study Group (2001). Prevention of type 2 diabetes mellitus by changes in lifestyle among subjects with impaired glucose tolerance. N Engl J Med.

[6] Knowler WC, Barrett-Connor E, Fowler SE, Hamman RF, Lachin JM, Walker EA (2002). Reduction in the incidence of type 2 diabetes with lifestyle intervention or metformin. N Engl J Med..

[7] Pan XR, Li GW, Hu YH, Wang JX, Yang WY, An ZX (1997). Effects of diet and exercise in preventing NIDDM in people with impaired glucose tolerance. The Da Qing IGT and Diabetes Study. Diabetes Care.

[8] Wareham NJ (2014). The long-term benefits of lifestyle interventions for prevention of diabetes. Lancet Diabetes Endocrinol..

[9] Li G, Zhang P, Wang J, An Y, Gong Q, Gregg EW (2014). Cardiovascular mortality, all-cause mortality, and diabetes incidence after lifestyle intervention for people with impaired glucose tolerance in the Da Qing Diabetes Prevention Study: a 23-year follow-up study. Lancet Diabetes Endocrinol..

[10] Neve M, Morgan PJ, Jones PR, Collins CE (2010). Effectiveness of web-based interventions in achieving weight loss and weight loss maintenance in overweight and obese adults: a systematic review with meta-analysis. Obes Rev..

[11] Wieland LS, Falzon L, Sciamanna CN, Trudeau KJ, Brodney S, Schwartz JE (2012). Interactive computer-based interventions for weight loss or weight maintenance in overweight or obese people. Cochrane Database Syst Rev.

[12] Brown MJ, Sinclair M, Liddle D, Hill AJ, Madden E, Stockdale J (2012). A systematic review investigating healthy lifestyle interventions incorporating goal setting strategies for preventing excess gestational weight gain. PLoS One.

[13] Pal K, Eastwood SV, Michie S, Farmer AJ, Barnard ML, Peacock R (2013). Computer-based diabetes self-management interventions for adults with type 2 diabetes mellitus. Cochrane Database Syst Rev.

[14] Franz MJ, Bantle JP, Beebe CA, Brunzell JD, Chiasson JL, Garg A (2002). Evidence-based nutrition principles and recommendations for the treatment and prevention of diabetes and related complications. Diabetes Care..

[15] International Society for DOHaD. What is DOHaD? 2014. https://dohadsoc.org/. Accessed 23 Oct 2015.

[16] Ge ZJ, Zhang CL, Schatten H, Sun QY (2014). Maternal diabetes mellitus and the origin of non-communicable diseases in offspring: the role of epigenetics. Biol Reprod..

[17] Inadera H (2013). Developmental origins of obesity and type 2 diabetes: molecular aspects and role of chemicals. Environ Health Prev Med..

[18] Hanson M, Gluckman PD, Ma RC, Matzen P, Biesma RG (2012). Early life opportunities for prevention of diabetes in low and middle income countries. BMC Public Health..

[19] American Diabetes Association (ADA) (2004). Gestational diabetes mellitus. Diabetes Care.

[20] Bartholow LK, Parcel GS, Kok G, Gottlieb NH (2006). Planning health promotion programs: an Intervention Mapping approach.

[21] Norris SA, Anuar H, Matzen P, Cheah JCH, Jensen BB, Hanson M. The life and health challenges of young Malaysian couples: results from a stakeholder consensus and engagement study to support non-communicable disease prevention. BMC Public Health. 2014; 14 Suppl 2. doi:10.1186/1471-2458-14-S2-S6. 10.1186/1471-2458-14-S2-S6 PMC4120157 25080995

[22] Institute for Public Health (IPH) (2011). National Health and Morbidity Survey 2011 (NHMS 2011). Vol. II: non-communicable diseases.

[23] Department of Statistics Malaysia. Population distribution and basic demographic characteristics report 2010 – Ethnic composition. 2010. [Online] https://www.statistics.gov.my/. Accessed 25 Nov 2015.

[24] Chan JM, Rimm EB, Colditz GA, Stampfer MJ, Willett WC (1994). Obesity, fat distribution, and weight gain as risk factors for clinical diabetes in men. Diabetes Care..

[25] Janssen I, Katzmarzyk PT, Ross R (2004). Waist circumference and not body mass index explains obesity-related health risk. Am J Clin Nutr..

[26] Brenner DR, Tepylo K, Eny KM, Cahill LE, El-Sohemy A (2010). Comparison of body mass index and waist circumference as predictors of cardiometabolic health in a population of young Canadian adults. Diabetol Metab Syndr..

[27] Eriksson J, Lindstrom J, Valle T, Aunola S, Hämäläinen H, Ilanne-Parikka P (1999). Prevention of type II diabetes in subjects with impaired glucose tolerance: the Diabetes Prevention Study (DPS) in Finland. Study design and 1-year interim report on the feasibility of the lifestyle intervention programme. Diabetologia..

[28] Crandall J, Schade D, Ma Y, Fujimoto WY, Barrett-Connor E, Fowler S (2006). The influence of age effects of lifestyle modification and metformin in prevention of diabetes. J Gerontol A Biol Sci Med Sci..

[29] Oldroyd JC, Unwin NC, White M, Imrie K, Mathers JC, Alberti KG (2001). Randomised controlled trial evaluating the effectiveness of behavioural interventions to modify cardiovascular risk factors in men and women with impaired glucose tolerance: outcome at 6 months. Diabetes Res Clin Pract..

[30] Bo S, Ciccone G, Baldi C, Benini L, Dusio F, Forastiere G (2007). Effectiveness of a lifestyle intervention on metabolic syndrome. A randomized controlled trial. J Gen Intern Med.

[31] Orozco LJ, Buchleitner AM, Gimenez-Perez G, Roque I, Figuls M, Richter B (2008). Exercise or exercise and diet for preventing type 2 diabetes mellitus. Cochrane Database Syst Rev.

[32] WHO STEPS Surveillance. Guide to physical measurements. 2008. http://www.who.int/chp/steps/manual/en/. Accessed 25 Nov 2015.

[33] HLS-EU Consortium. Comparative report of health literacy in eight EU member states. The European health literacy survey HLS-EU. 2012. http://www.health-literacy.eu. Accessed 25 Nov 2015

[34] Lovibond PF, Lovibond SH (1995). The structure of negative emotional states: comparison of the Depression Anxiety Stress Scales (DASS) with the Beck Depression and Anxiety Inventories. Behav Res Ther.

[35] Henry JD, Crawford JR (2005). The short-form version of the Depression Anxiety Stress Scales (DASS-21): construct validity and normative data in a large non-clinical sample. Br J Clin Psychol..

[36] Koivusalo SB, Rono K, Klemetti MM, Roine RP, Lindström J, Erkkola M (2015). Gestational diabetes mellitus can be prevented by lifestyle intervention: The Finnish Gestational Diabetes Prevention Study (RADIEL). Diabetes Care.

[37] Guelinckx I, Devlieger R, Mullie P, Vansant G (2014). Effect of lifestyle intervention on dietary habits, physical activity, and gestational weight gain in obese pregnant women: a randomised controlled trial. Am J Clin Nutr..

[38] Quinlivan JA, Lam LT, Fisher J (2011). A randomised trial of a four-step multidisciplinary approach to the antenatal care of obese pregnant women. Aust N J Obstet Gynaecol..

[39] Phelan S, Phipps MG, Abrams R, Darroch F, Schaffner A, Wing RR (2011). Randomized trial of a behavioural intervention to prevent excessive gestational weight gain: The fit for delivery study. Am J Clin Nutr..

[40] Vinter CA, Jensen DM, Ovesen P, Beck-Nielsen H, Jørgensen JS (2011). The Lip (Lifestyle in Pregnancy) study: a randomized controlled trial of lifestyle intervention in 360 obese pregnant women. Diabetes Care..

[41] Whitworth M, Dowswell T (2009). Routine pre-pregnancy health promotion for improving pregnancy outcome. Cochrane Database Syst Rev.

[42] Shawe J, Delbarere I, Ekstrand M, Hegaard HK, Larsson M, Mastroiacovo P (2014). Preconception care policy, guidelines, recommendations and services across six European countries: Belgium (Flanders), Denmark, Italy, the Netherlands, Sweden and the United Kingdom. Eur J Contracept Reprod Health Care.

